# Identification of phenological QTLs using a combination of high- and low-coverage whole genome sequencing in Japanese plum (*Prunus salicina* Lindl.)

**DOI:** 10.1093/hr/uhaf271

**Published:** 2025-11-04

**Authors:** María Nicolás-Almansa, David Ruiz, Alfonso Guevara, Manuel Rubio, Pedro Martínez-Gómez, Pat J Brown, Pedro J Martínez-García

**Affiliations:** Department of Plant Breeding, CEBAS-CSIC, PO Box 164, E-30100 Espinardo, Murcia, Spain; Department of Applied Biology, Escuela Politécnica Superior de Orihuela, University Miguel Hernández, Ctra. Beniel km. 3.2, Orihuela, 03312 Alicante, Spain; Department of Plant Breeding, CEBAS-CSIC, PO Box 164, E-30100 Espinardo, Murcia, Spain; Department of Hortofruticulture, IMIDA, C/ Mayor s/n, 30150 La Alberca, Murcia, Spain; Department of Plant Breeding, CEBAS-CSIC, PO Box 164, E-30100 Espinardo, Murcia, Spain; Department of Plant Breeding, CEBAS-CSIC, PO Box 164, E-30100 Espinardo, Murcia, Spain; Department of Plant Sciences, University of California, Davis, CA 95616, USA; Department of Plant Breeding, CEBAS-CSIC, PO Box 164, E-30100 Espinardo, Murcia, Spain

## Abstract

The genetic control of phenological traits in Japanese plum (*Prunus salicina* Lindl.) was investigated through quantitative trait loci (QTL) analysis in three segregating F_1_ populations: ‘Black Splendor’ × ‘Pioneer’ (BS×PIO), ‘Red Beaut’ × ‘Black Splendor’ (RB×BS), and ‘Red Beaut’ × ‘Santa Rosa Precoz’ (RB×SRP), comprising 121, 103, and 103 seedlings, respectively. Whole-genome sequencing (~80×) was conducted for the four parents, and progenies were genotyped using a cost-efficient reduced-representation sequencing strategy. SNPs heterozygous in one parent and homozygous in the other were used to build six parental linkage maps. Phenological traits, including beginning, full, and end of flowering (BF, FF, EF), flowering intensity (FI), ripening date (RD), fruit development period (FDP), and productivity (P), were evaluated over three years. A total of 53 QTLs were identified for flowering stages, 16 for RD, 18 for FDP, 10 for FI, and 16 for P. Many QTLs were stable across years. Major QTLs for flowering traits were mapped to LG1, LG2, LG4, and LG6, with a strong QTL for FF on LG6 of ‘Black Splendor’. In BS×PIO, BF was uncorrelated with FF and EF, indicating distinct genetic control likely inherited from ‘PIO’, a low-chill cultivar. RD and FDP were consistently associated with LG4, while productivity QTLs were detected on LG1, LG2, and LG4, often overlapping, suggesting pleiotropic or tightly linked loci. In addition, candidate genes within stable QTLs were detected, providing immediate targets for functional studies. This study provides one of the first genome-wide QTL analyses of phenology in Japanese plum using low-coverage whole genome sequencing and offers valuable tools for marker-assisted breeding in this species.

## Introduction

The *Prunus* genus encompasses economically significant species in global agriculture, including *P. persica* (peach), *P. avium* (sweet cherry), *P. dulcis* (almond), *P. armeniaca* (apricot) and plum species. The plum species, Japanese plum (*Prunus salicina* Lindl.) and European plum (*P. domestica* L.), rank as the second most important stone fruit crop following peaches, with a worldwide production of around 12 million tons [1]. Unlike the hexaploid European plum (6*n* = 2*x* = 48), the Japanese plum has a diploid genome (2*n* = 2*x* = 16). It originated in China, but its modern breeding history began in California by Luther Burbank, who introduced interspecific crosses with several diploid plums, such as *P. americana* Marshall, *P. hortulana* L. H. Bailey, *P. munsoniana* W. Wight & Hedrick, or *P. simonii* Carrière, to enhance its adaptation to local environments. Consequently, the term ‘Japanese plum’ refers to a diverse group of hybrids derived from crosses involving up to 15 different *Prunus* species rather than a single pure species [2].

Similar to other *Prunus* species and woody crops, plums typically have long breeding cycles due to extended juvenile periods, complex reproductive biology, and a high degree of heterozygosity [3]. The development of new cultivars is particularly needed in plums, as global production largely depends on a limited number of heirloom cultivars [4]. The improved cultivars can compensate for the shortcomings of these heirloom cultivars. While the most advanced plum breeding programs are primarily based in California [2], significant efforts are also underway in countries such as China, Japan, Chile, and Spain [5, 6]. The improvement of phenological traits, such as flowering time or ripening date, remains one of the main breeding objectives [2, 7], but these traits are also increasingly affected by climate change [8, 9]. In regions such as Badajoz, Spain, reduced winter chill threatens dormancy release and flowering stability [10, 11].

The use of biotechnological approaches, such as marker-assisted selection (MAS) or genomics, can help accelerate the highly time-consuming breeding process in plum to develop climate-resilient cultivars. The availability of molecular markers in Japanese plum remains limited, with initial efforts focused primarily on self-incompatibility markers, which support the evaluation of inter-compatibility among genotypes and aid in crossbreeding strategies [12]. More recently, markers associated with skin color [13] and flesh color [14] have been developed, enabling precise prediction of red pigmentation in both tissues.

Compared to other *Prunus* species, only a few studies have focused on the identification of quantitative trait loci (QTLs) linked to phenological and fruit quality traits in Japanese plum [15, 16]. These studies have identified QTLs for traits such as flowering time, ripening date, and flavonoid content, primarily using linkage mapping based on genotyping-by-sequencing (GBS). The main drawback of these studies is that they relied on the peach genome [17] as a reference for read mapping, relying on the high synteny between *Prunus* species [18].

More recently, the release of a new reference genome for *P*. *salicina* has allowed the improvement of these previous results [19]. These authors employed the genome of cv. ‘Sanyueli’ [20] to improve previous linkage maps and to elucidate QTLs associated with phenolic compound content, specifically flavan-3-ols. Although, GBS is a relatively inexpensive method for genotyping large numbers of samples and provides more SNPs than SNP arrays [21], new applications, also based on the reduced representation of the genome, have been developed in the last ten years, which greatly reduce the cost of sequencing [22]. Another cost-effective solution for F_1_ populations typically used in fruit tree breeding was recently proposed [23]. These authors suggested that the use of the Smooth Descent (SD) algorithm, with long-read sequencing of the parents and low-depth sequencing with short reads of the descendants would reduce genotyping costs. However, the application of these new approaches is clearly scarce in Japanese plum and in *Prunus* species in general.

The main goal of this study was to investigate the genetic control of key phenological traits in Japanese plum through QTL analysis of three F_1_ populations. We aimed to construct linkage maps and identify stable QTLs and candidate genes using an integrated whole-genome sequencing strategy.

## Results

### Genome sequencing and assembly

The total number of raw reads was 720 391 200, with the highest number obtained for ‘SRP’ (193383502) and the lowest for ‘Black Splendor’ (174744788). The total data output from the sequencer was 108.1 Gb. After trimming the raw reads, more than 89% of the raw data were retained. The highest number of retained reads was observed for BS, with 90.03% (157 321 936) (Table S2).

The lengths of the assembled genomes ranged from 256.42 Mb for SRP to 239.11 Mb for BS. Overall, a relatively small N50 value was observed for all genomes, with BS exhibiting the highest N50, indicating a higher proportion of longer contigs. The GC content was consistent across all genomes, averaging approximately 37%. The SRP genome had the highest number of contigs (97 639), reflecting greater fragmentation, while the BS genome contained the fewest (81 968). However, BS also had the largest contig, suggesting better representation of long continuous sequences. Furthermore, BS exhibited a greater total length of long contigs (≥10 000 bp and ≥ 25 000 bp), indicating that despite having fewer contigs, they are substantially longer. All assemblies showed zero ambiguous sequences, indicating a high level of precision in the genome assembly process (Table S3).

**Figure 1:**
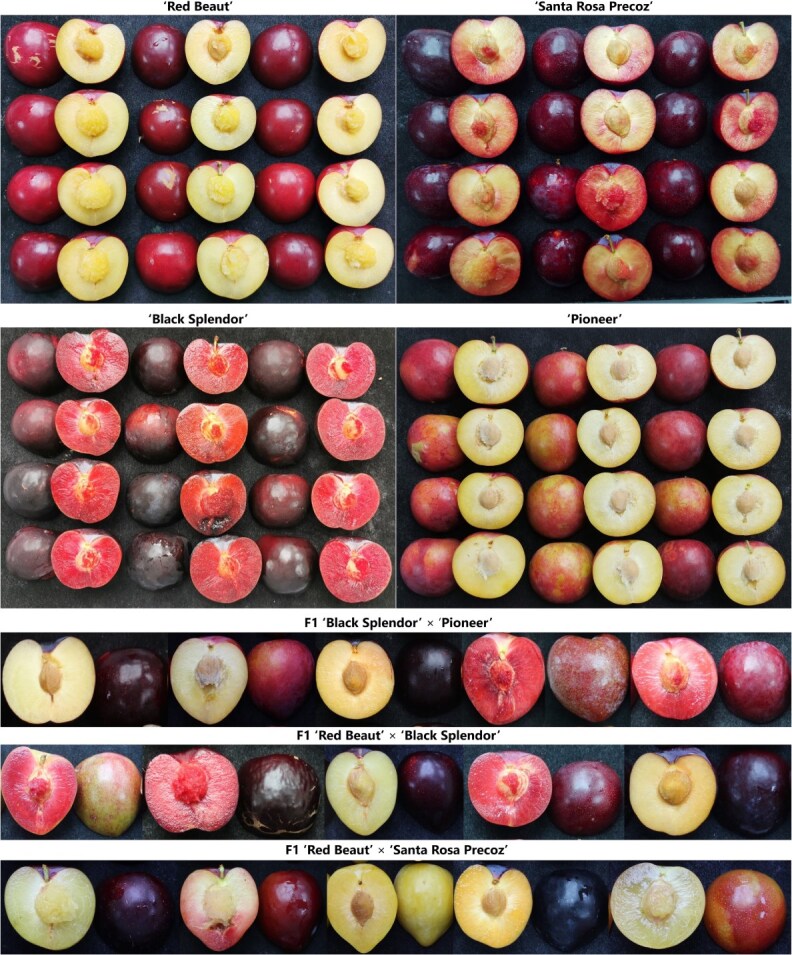
Illustrative images of several fruits of each parental genotype and fruits of each population evaluated in this study.

**Figure 2:**
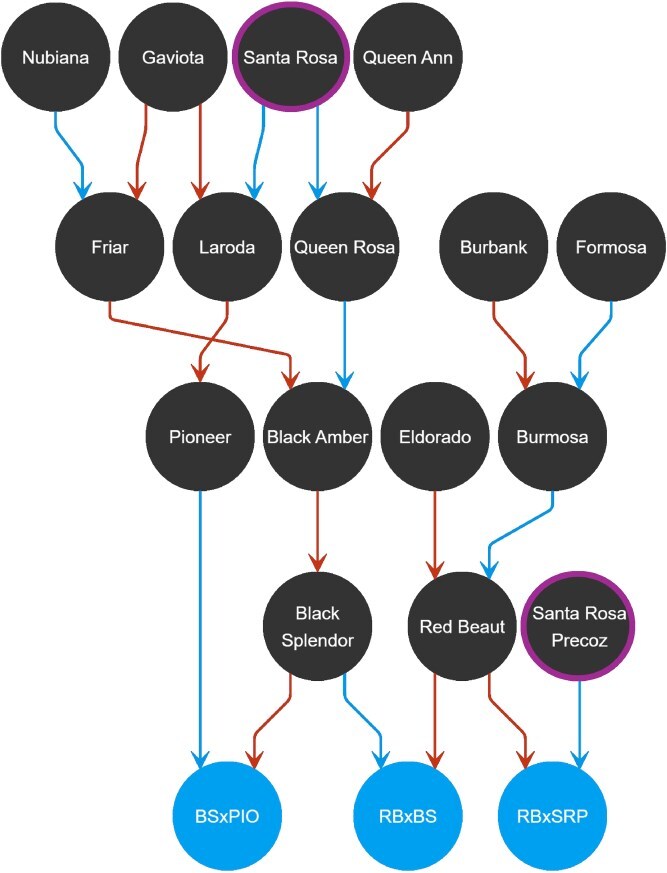
The complete pedigree for each parental genotype. Magenta circle highlights ‘Santa Rosa’ and its spontaneous mutant ‘Santa Rosa Precoz’, identified by CSIC technician A. Molina [24]. In blue are the three F_1_ Japanese plum populations.

After quality control, reads were realigned to the genome assemblies to assess similarity. All reads from the four parental assemblies were paired, with SRP showing the highest number of paired reads (96.7 million), followed by PIO (88.5 million), RB (87.6 million), and BS (87.4 million). The assemblies exhibited high alignment quality, with approximately 50% of reads aligning consistently at the correct distance and orientation to the MaSuRCA assemblies. Notably, the PIO and BS assemblies had the highest exact alignment rates (PIO: 51.61%; BS: 52.06%) and the highest overall alignment rate (94.34%) (Table S4).

The BUSCO analysis, based on the set of single-copy orthologs, showed that more than 80% of the expected genes were identified as complete (81.9% in SRP, 84.5% in PIO, 83.6% in RB, and 84.9% in BS) for the MASURCA assembly (Table S5).

### Genetic variation analysis in the parental genotypes

Regarding small genetic variation, a total of 9 223 636 genetic variants were identified using the analytical pipeline employed in this study. This dataset included 276 491 deletions, 284 641 small insertions, and 8 662 504 SNPs (Table S6). To refine the dataset, SNPs with two or more alternative alleles in the VCF file were excluded, resulting in a final set of 8 616 483 SNPs. This filtered set was subsequently used to calculate the number of transitions and transversions. Within this final SNP dataset, 59% were transitions (5 067 800 loci) and 41% were transversions (3 548 683 loci), yielding a transition/transversion (Ts/Tv) ratio of 1.44 for SRP, 1.43 for PIO, 1.43 for RB, and 1.41 for BS. Among the transition variants, A/G and C/T substitutions were the most frequent and occurred at similar rates. Among transversions, A/T substitutions exhibited the highest frequency (~32%), whereas C/G substitutions were the least frequent (~16%) (Table S7).

**Figure 3:**
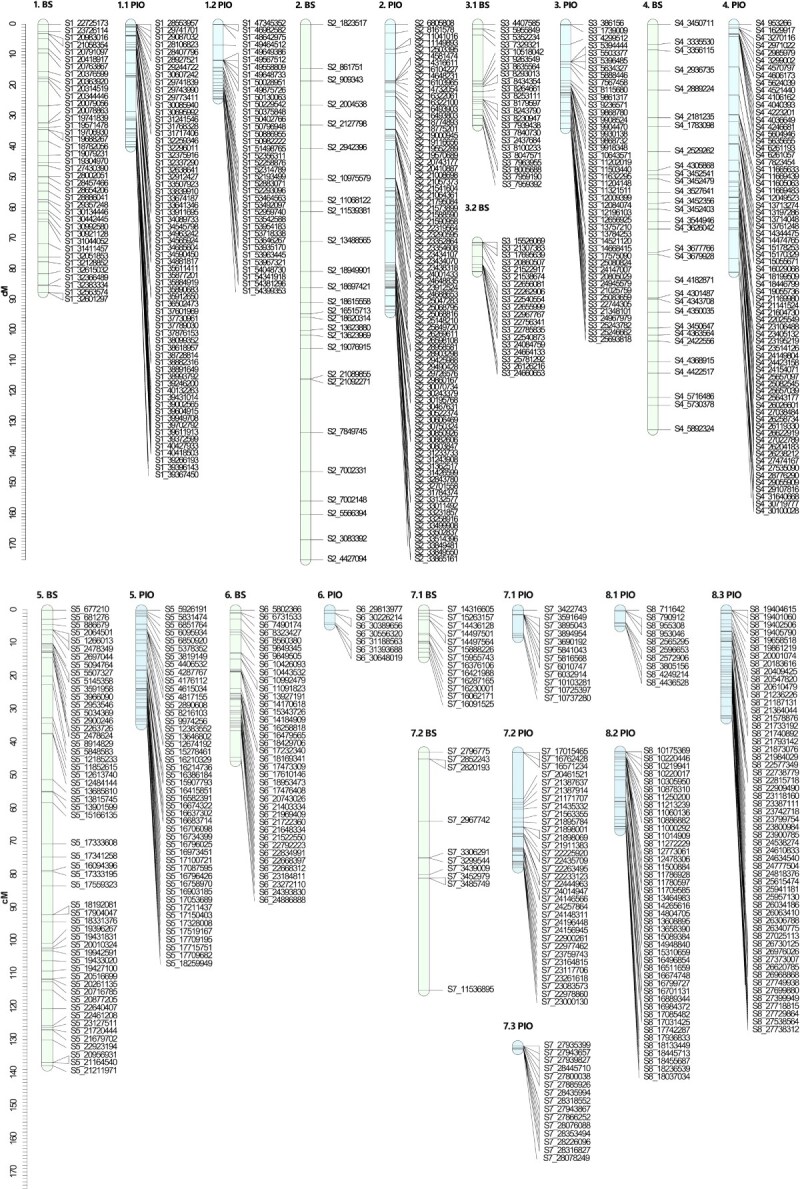
Genetic linkage maps for each genitor in the F_1_ Japanese plum population ‘Black Splendor’ × ‘Pioneer’. Markers positions are represented by black lines. The linkage groups of the female progenitor ‘Black Splendor’ (BS) are indicated in light blue, and those of the male progenitor ‘Pioneer’ (PIO) are indicated in green.

### Genotypic characterization of the offspring

Two independent reduced representation libraries were prepared for each DNA sample using two different restriction enzymes, HindIII and PstI. The average number of barcoded reads per sample was similar between both restriction enzymes, with a mean of 339 194 for HindIII and 329 254 for PstI. Likewise, the proportion of barcoded reads that successfully aligned to the plum reference genome was comparable, reaching 87.4% for HindIII and 89.5% for PstI.

Of the 384 DNA samples analyzed, 11 (2.9%) had fewer than 10 000 reads in both libraries, indicating low DNA quality. Additionally, seven samples (1.8%) had fewer than 10 000 reads only in the PstI library, whereas 24 samples (6.3%) had fewer than 10 000 reads exclusively in the HindIII library. These findings suggest that the PstI library preparation protocol may be slightly more robust than HindIII. The number of raw SNPs identified per haplotype of each population was at least twice as high in the HindIII library compared to the PstI library (Table S8).

SNPs from both libraries were pooled and depth-filtered (genotypes with depth less than 5 were set to missing) before imputation with FSFHap. This process led to the loss of a substantial number of low-coverage SNPs. Of the 48 chromosomes targeted for imputation (8 plum chromosomes × 3 populations × 2 parents), two failed: chromosome 8 of ‘Black Splendor’ could not be imputed in either the BS × PIO or RB × BS populations, likely due to the low number of raw SNPs in these regions. We infer that the ‘Black Splendor’ cultivar may be identical-by-descent for portions of chr8.

### Transmission and correlations of phenological traits

The results showed that none of the traits followed a normal distribution pattern within any of the three families (Table S9). Notably, a significant proportion of seedlings across the three progenies exhibited transgressive values beyond those of their parents for the most of traits (Figs S1–S3).

The BS × PIO progeny exhibited the earliest flowering among the studied populations, with a considerable number of individuals flowering earlier than both parental genotypes, BS and PIO. Depending on the year, full flowering in some seedlings occurred between January 20 and February 5 (Julian days 20 to 36) (Fig. S1, Fig. S4A, Table S9). In contrast, the progenies of RB × BS and RB × SRP initiated flowering in mid-February, between Julian days 41 and 47. The majority of individuals in both populations exhibited delayed flowering compared to their respective parental genotypes (Fig. S2, Fig. S4A, Table S9). Notably, the RB × SRP progeny exhibited the latest flowering time among the studied populations. Most genotypes within this progeny reached full flowering starting from February 25 (Julian day 55) and extending into the first or second week of March, depending on the year (Fig. S3, Fig. S4A, Table S9).

Regarding flowering intensity and productivity, most individuals from the three progenies exhibited lower values than their respective parents, indicating the presence of hybrid weakness for these traits. This is consistent with the fact that the parental genotypes of all three families displayed high flowering intensity and productivity (Figs S1–S3, Table S9). Nevertheless, the BS × PIO population showed higher flowering intensity and productivity compared to the other two progenies, while RB × SRP exhibited the lowest values overall. In addition, the BS × PIO progeny exhibited a markedly extended fruit development period, lasting approximately five months (Fig. S1, Fig. S4A, Table S9).

The Kruskal–Wallis test revealed significant differences among years for all evaluated traits, except for productivity and flowering intensity, which did not show significant differences in any of the populations (Table S10). Flowering dates differed significantly between years in all progenies, except for flowering onset in the BS × PIO population. According to the pairwise comparison analysis (Table S11), significant differences were found for flowering dates, except between 2019 and 2021 for full bloom and end of flowering in the BS × PIO progeny.

Significant correlations among phenological traits were observed across all three progenies over the three-year study (Fig. S5). In RB × BS and RB × SRP populations, flowering dates (BF, FF, EF) showed strong correlations (*r* > 0.74^***^), whereas in BS × PIO, only FF and EF were strongly correlated (*r* > 0.93^***^). Flowering intensity (FI) and productivity (P) were positively correlated in RB × BS (*r* = 0.62^***^) and RB × SRP (*r* = 0.59^***^). High correlations (*r* > 0.90^***^) were also found between FM and FDP in both populations. Notably, inverse correlations between FM/FDP and P, and between flowering dates and FDP, were observed in RB × BS, with FF in 2021 showing the strongest correlation (*r* = −0.47^*^). In RB × SRP, flowering dates and FM were significantly correlated, especially in 2019 and 2020. Interannual trait consistency was also high (Table S12).

The strongest year-to-year correlations for flowering dates were seen in BS × PIO (*r* > 0.77^***^), followed by RB × BS (*r* > 0.65^***^) and RB × SRP (*r* > 0.51^***^). FI showed high interannual stability in RB × BS (*r* = 0.99^***^), RB × SRP (*r* > 0.86^***^), and to a lesser extent in BS × PIO (*r* > 0.41^***^). RD, FDP, and P exhibited consistently high correlations across years in all progenies (*r* > 0.81^***^) (Table S12).

Principal component analysis (PCA) of phenological traits (Fig. S4A) revealed key patterns of variation among the plum progenies. The first two components, PC1 and PC2, explained 37.8% and 22.4% of the total variance, respectively. The scree plot (Fig. S4B) showed that the first four components together accounted for over 80% of the observed variance. Flowering traits (BF, FF, EF) were the main contributors to PC1, while RD and FDP predominantly influenced PC2. PC3 was primarily shaped by FI and P, highlighting their distinct contribution to phenotypic variation.

### Linkage mapping

Genetic linkage maps were independently constructed for each parent across the three F_1_ Japanese plum populations using SNP markers filtered for quality (biallelic, MAF > 0.05, and less than 40% missing data) (Table S13).

In the BS × PIO population, 252 SNPs were mapped for the Black Splendor (BS) parent and 519 for the PIO parent, resulting in 9 linkage groups (LGs) for BS and 13 for PIO (Fig. 3). The BS map spanned 707.78 centimorgans (cM), with LG2 as the longest (175.08 cM), while PIO had a shorter map (417.64 cM) with higher marker density (0.71 cM per SNP) and fragmentation in LG1, LG7, and LG8 (Table 1).

**Table 1:** Description of each linkage group (LG) for each genitor in the F_1_ Japanese plum population ‘Black Splendor’ × ‘Pioneer’.

	**LG**	**SNPs**	**Total distance (cM)**	**SNP density (cM/SNP)**	**Maximum gap (cM)**
BS	1	39	87.71	2.25	8.01
	2	26	175.08	6.73	17.38
	3.1	23	33.17	1.44	15.05
	3.2	19	9.67	0.51	3.72
	4	30	132.61	4.42	9.07
	5	54	137.75	2.55	13.83
	6	38	45.56	1.20	7.95
	7.1	13	14.18	1.09	4.11
	7.2	10	72.05	7.20	33.93
	8	0			
	Total	252	707.78	3.04	
PIO	1.1	64	40.08	0.63	11.37
	1.2	38	24.36	0.64	6.71
	2	82	94.32	1.15	10.41
	3	45	34.06	0.76	6.30
	4	69	81.11	1.18	10.00
	5	46	34.47	0.75	3.15
	6	7	4.29	0.61	2.86
	7.1	12	8.08	0.67	5.62
	7.2	33	34.78	1.05	14.01
	7.3	15	0.85	0.06	0.42
	8.1	10	4.84	0.48	3.44
	8.2	43	23.69	0.55	5.29
	8.3	55	32.73	0.60	2.92
	Total	519	417.64	0.71	

**Figure 4:**
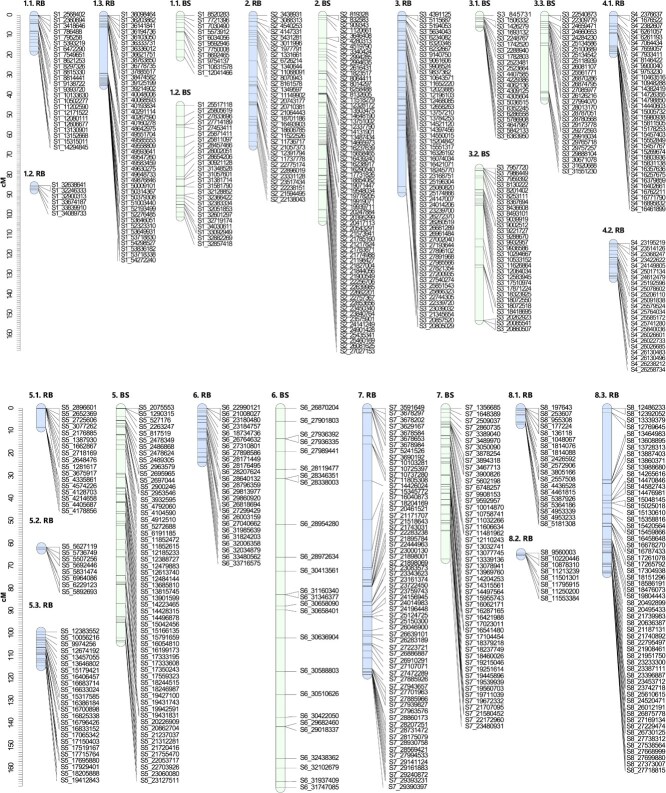
Genetic linkage maps for each genitor in the F_1_ Japanese plum population ‘ Beaut’ × ‘Black Splendor’. Markers positions are represented by black lines. The linkage groups of the female progenitor ‘Red Beaut’ (RB) are indicated in light blue, and those of the male progenitor ‘Black Splendor’ (BS) are indicated in green.

For the RB × BS population, 441 SNPs were mapped for the RB parent and 334 for BS (Fig. 4). The RB map comprised 15 LGs with multiple unlinked segments in LG1, LG4, and LG8, covering 494.79 cM. In contrast, BS displayed 9 LGs spanning 769.81 cM, with lower marker density (2.17 cM per SNP) (Table 2).

**Figure 5:**
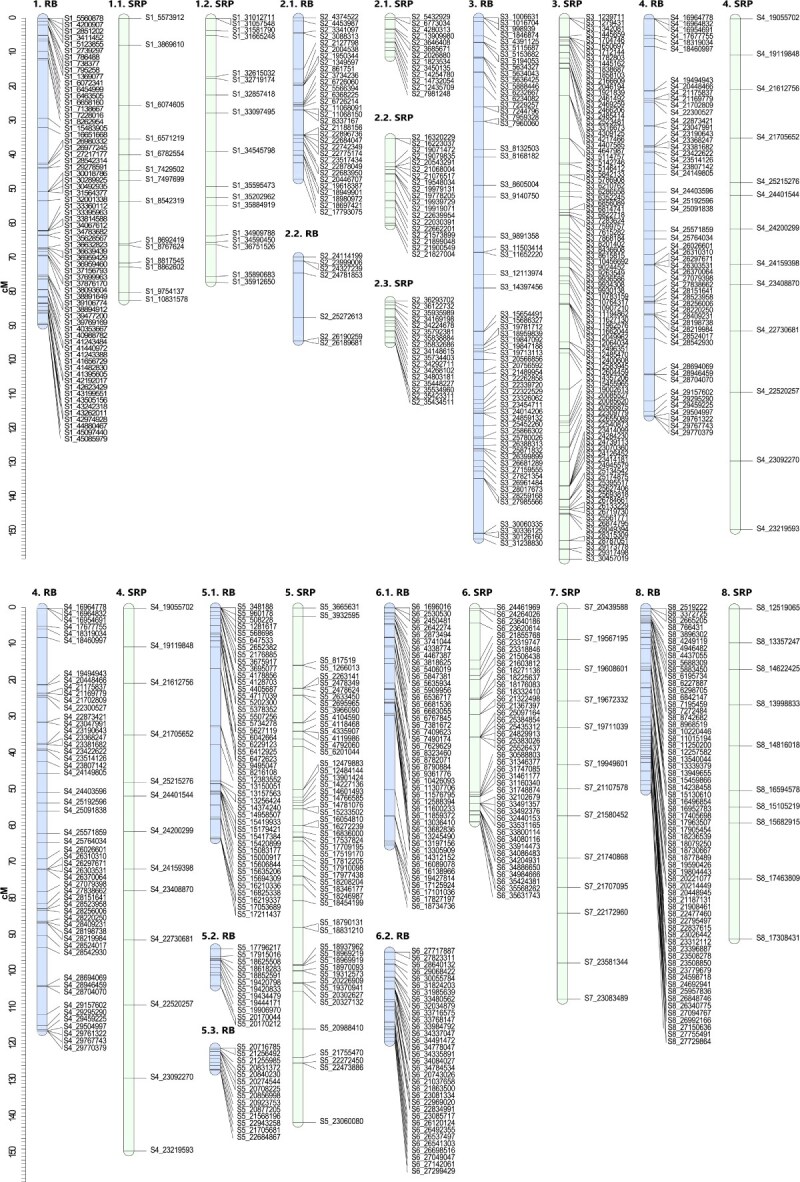
Genetic linkage maps for each genitor in the F_1_ Japanese plum population ‘Red Beaut’ × ‘Santa Rosa Precoz’. Markers positions are represented by black lines. The linkage groups of the female progenitor ‘Red Beaut’ (RB) are indicated in light blue, and those of the male progenitor ‘Santa Rosa Precoz’ (SRP) are indicated in green.

In the RB × SRP population, 428 SNPs were mapped for RB and 305 for SRP (Fig. 5). The RB map had 11 LGs, including segmented LG2, LG5, and LG6, and spanned 646.26 cM. The SRP map extended to 909.23 cM with lower marker density (4.37 cM per SNP) and a large gap in LG4 (20.08 cM) (Table 3).

### Identification of QTLs linked to phenological traits

Using the Kruskal–Wallis test, a total of 113 QTLs were identified across the three F_1_ families for the phenological traits evaluated, of which 60 were considered stable.

#### Flowering phases (BF, FF, EF)

**Figure 6:**
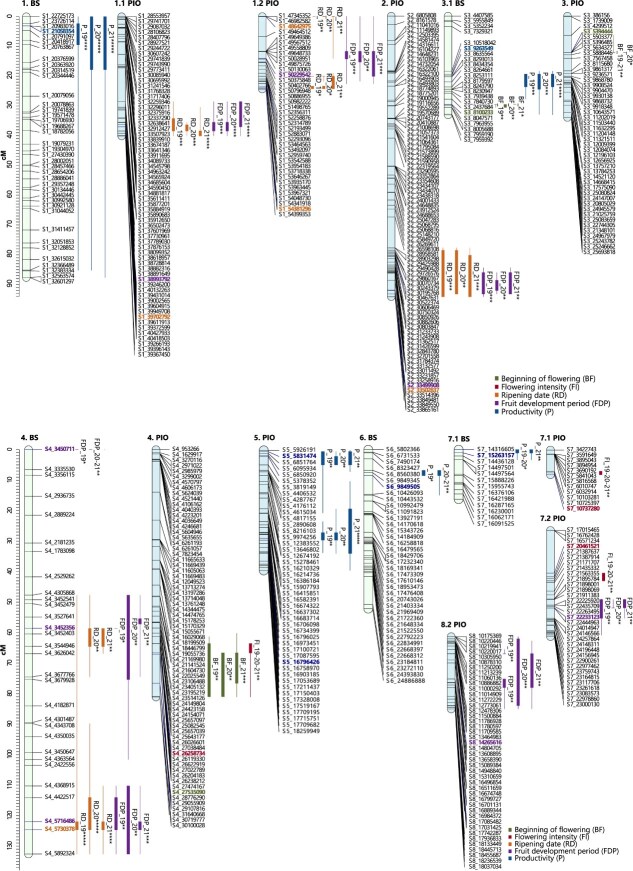
Consistent QTLs across the three years of phenotyping for phenological traits in the parents of the ‘Black Splendor’ (BS) × ‘Pioneer’ (PIO) population. The first number indicates the scaffold it represents, while the second number indicates the subgroup when multiple linkage groups are involved. The markers are listed on the right side of each LG, and the QTLs are depicted on the left side of their corresponding LGs. The representation of the QTLs includes a thick line indicating the most significant part of the QTL and a thin line indicating the entire significant interval (^*^P < 0.01; ^**^P < 0.005; ^***^P < 0.001; ^****^P < 0.0005; ^*****^P < 0.0001). The most significant markers of each QTL are shown in bold and in the color assigned to the trait.

**Figure 7:**
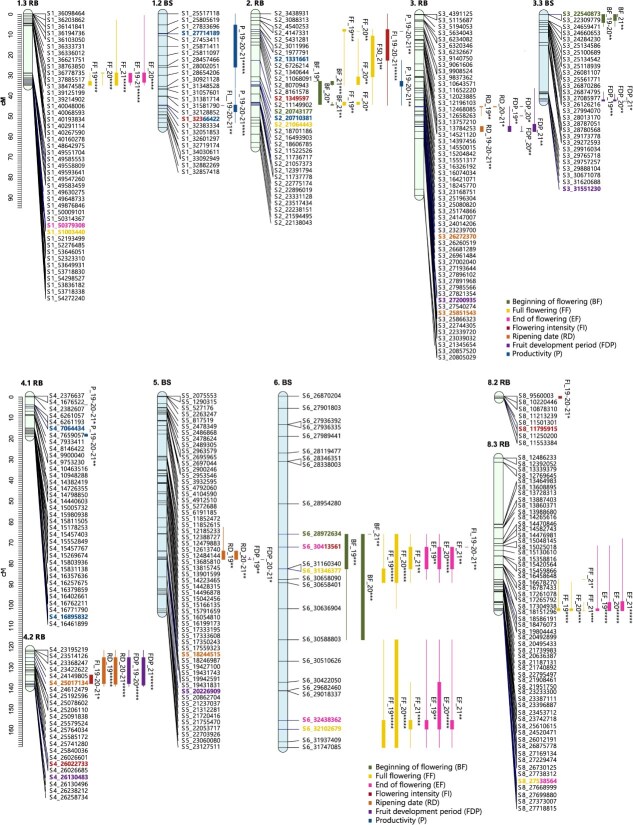
Consistent QTLs across the three years of phenotyping for phenological traits in the parents of the ‘Red Beaut’ (RB) × ‘Black Splendor’ (BS) population. The first number indicates the scaffold it represents, while the second number indicates the subgroup when multiple linkage groups are involved. The markers are listed on the right side of each LG, and the QTLs are depicted on the left side of their corresponding LGs. The representation of the QTLs includes a thick line indicating the most significant part of the QTL and a thin line indicating the entire significant interval (^*^P < 0.01; ^**^P < 0.005; ^***^P < 0.001; ^****^P < 0.0005; ^*****^P < 0.0001). The most significant markers of each QTL are shown in bold and in the color assigned to the trait.

**Figure 8:**
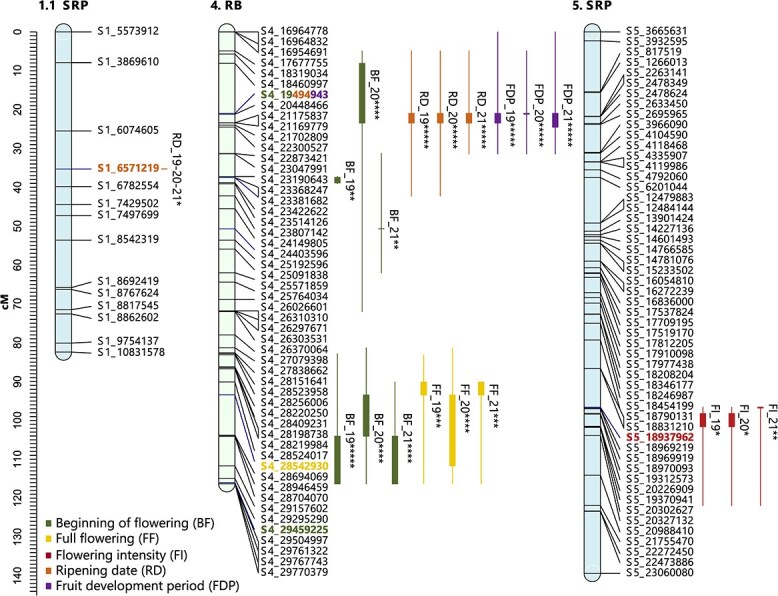
Consistent QTLs across the three years of phenotyping for phenological traits in the parents of the ‘Red Beaut’ (RB) × ‘Santa Rosa Precoz’ (SRP) population. The first number indicates the scaffold it represents, while the second number indicates the subgroup when multiple linkage groups are involved. The markers are listed on the right side of each LG, and the QTLs are depicted on the left side of their corresponding LGs. The representation of the QTLs includes a thick line indicating the most significant part of the QTL and a thin line indicating the entire significant interval (^*^P < 0.01; ^**^P < 0.005; ^***^P < 0.001; ^****^P < 0.0005; ^*****^P < 0.0001). The most significant markers of each QTL are shown in bold and in the color assigned to the trait.

A total of 53 QTLs were detected—22 for full flowering (FF), 17 for end of flowering (EF), and 14 for beginning of flowering (BF), with 18 classified as stable (Tables S14–S16). The RB × BS population contributed the most QTLs (32 total, 12 stable), followed by RB × SRP (15 total, 3 stable) and BS × PIO (6 total, 3 stable). Stable QTLs were mainly located on LG6 (5 QTLs), LG4 (4), LG3 (3), and LG2 (2) (Figs 6–8). Most SNPs associated with stable QTLs linked to different flowering dates exhibited a significant allelic effect over the three years of study, predominantly associated with a delayed flowering time (Tables S17, S18 and S19). Notably, SNP S6_32102679 on LG6 (in BS, RB × BS family) was highly associated with FF (KW = 24.05, p < 0.0001) (Table S18). Individuals carrying the ‘T’ allele showed delayed FF compared to those with ‘CC’ genotype (Fig. S6). In contrast, for SNP S3_22540873 (LG3.3, BS in RB × BS), the ‘G’ allele was linked to earlier BF (Fig. S7).

#### Flowering intensity

Ten QTLs were associated with flowering intensity (FI), of which nine were stable. RB × BS and BS × PIO each contributed 5 and 3 stable QTLs, respectively, while RB × SRP contributed two (one stable). Stable QTLs were distributed across most LGs except LG3 and LG8 (Tables S14–S16). In RB × BS, the most significant QTL was on LG2 (RB), with SNP S2_1349597 showing that the ‘C’ allele increased flowering intensity (Fig. S8). For PIO (BS × PIO), stable QTLs were found on LG4 and LG7. SNPs such as S4_26258734 and S7_10737280 were associated with higher FI, while S7_20461521 showed a negative effect of the ‘A’ allele (Table S17).

#### Ripening date and fruit development period

Sixteen QTLs were identified for ripening date (RD) (11 stable), and 18 for fruit development period (FDP) (12 stable). BS × PIO and RB × BS contributed the most stable QTLs. Stable RD QTLs were mostly located on LG1 and LG4.The most significant RD QTL was found on LG4.2 of RB (RB × BS) (Table S15), with SNP S4_25017134 showing earlier ripening in ‘A’ allele carriers (Table S18). Another RD QTL on LG4 in RB (RB × SRP) involved SNP S4_19494943, where the ‘T’ allele delayed ripening (Fig. S9, Table S19). For FDP, LG4 showed the highest number of stable QTLs. The most significant FDP QTL overlapped with the RD QTL above, with the ‘T’ allele of S4_19494943 linked to longer development (Table S19).

#### Productivity

Sixteen QTLs were detected for productivity, 10 of which were stable. BS × PIO contributed the most (9 QTLs, 6 stable), followed by RB × BS (4 stable QTLs). No stable QTLs were found in RB × SRP. Stable QTLs were distributed across LG1 to LG7 (except LG8), with LG2, LG4, and LG5 contributing the highest numbers (Tables S14–S16). The most significant productivity QTL was located on LG1 of BS (BS × PIO), where SNP S1_21058354 showed that the ‘G’ allele conferred higher productivity compared to the ‘AA’ genotype (Table S17).

**Table 2:** Description of each linkage group (LG) for each genitor in the F_1_ Japanese plum population ‘Red Beaut’ × ‘Black Splendor’.

	**LG**	**SNPs**	**Total distance (cM)**	**SNP density (cM/SNP)**	**Maximum gap (cM)**
RB	1.1	24	18.02	0.75	3.85
	1.2	6	2.14	0.36	0.63
	1.3	44	35.09	0.80	23.06
	2	33	65.54	1.99	8.12
	3	55	88.92	1.62	24.80
	4.1	35	19.24	0.55	5.98
	4.2	23	17.50	0.76	2.99
	5.1	17	8.52	0.50	8.40
	5.2	8	0.99	0.12	0.59
	5.3	24	15.17	0.63	1.58
	6	25	23.98	0.96	3.50
	7	63	118.35	1.88	17.28
	8.1	19	6.99	0.37	2.71
	8.3	57	73.34	1.29	29.70
	8.2	8	1.00	0.12	0.55
	Total	441	494.79	0.85	
BS	1.1	11	20.88	1.90	4.55
	1.2	25	55.83	2.23	5.43
	2	64	103.24	1.61	9.69
	3.1	22	6.00	0.27	1.94
	3.2	29	76.20	2.63	26.49
	3.3	28	42.53	1.52	22.77
	4	0			
	5	58	103.94	1.79	10.65
	6	47	168.80	6.26	21.80
	7	50	67.04	1.34	12.56
	8	0			
	Total	334	769.81	2.17	

## Discussion

This study presents one of the first comprehensive genome-wide analyses of phenological traits in Japanese plum (*P. salicina*), integrating high-coverage WGS of parents and low-coverage WGS (lcWGS) of progenies to construct linkage maps and identify QTLs associated with key traits. This hybrid strategy can help reduce some challenges associated with lc-WGS such as genotype misclassification or sequencing errors being erroneously classified as genetic variants [46]. In addition, this strategy proved to be highly cost-effective and well suited for implementation in new breeding programs. The total genotyping costs for the progeny were maintained below $6 per individual. These costs are consistent with other cost-efficient methods, such as Skim-seq, which reports per-sample genotyping costs below $3 [47]. In terms of cost per sample, and for the construction of genetic maps in medium-size breeding populations, this strategy is considerably cheaper than traditional SNP arrays; for example, the 60 K array developed for almond costs approximately 33 € per sample, even for consortium members (H. Duval pers. comm). Other strategies such as GBS typically around $30 per sample depending on depth and multiplexing level [34].

In this study, we present, for the first time, genetic linkage maps for Japanese plum constructed using the *P. salicina* ‘Sanyueli’ reference genome. A limited number of genetic linkage maps have been published for Japanese plum, all derived from F_1_ populations (Table S20). Among the maps generated, ‘SRP’ yielded the longest (909.23 cM) with a marker density of 4.37 cM per marker, except for the ‘SR’ male parent in [48], which reached 1349.6 cM albeit with lower marker resolution (16.1 cM/marker). However, all these previous maps were aligned to the peach reference genome. More recently, Battistoni *et al.* [19] enhanced the maps of ‘98–99’ and ‘Angeleno’ by aligning them to the *P. salicina* ‘Sanyueli’ v2.0 reference genome. Overall, the genetic maps developed in this study demonstrate high quality and resolution, while being generated through a cost-effective approach, making them valuable resources for Japanese plum breeding programs.

Phenological evaluation across the three progenies revealed broad variability, with many seedlings displaying transgressive values beyond parental ranges. This reflects the impact of genetic background, hybridization, and self-incompatibility in enhancing heterozygosity and generating phenotypic extremes in Japanese plum [51, 52]. Such diversity also explains the negative heterosis observed for flowering intensity and productivity, where most offspring underperformed compared to parents—a trend consistent with other *Prunus* species like sweet cherry and almond [53, 54]. Additionally, productivity may have been influenced by the use of commercial rootstocks in parental trees, known to affect vigor and yield [55]. Similar transgressive patterns have been reported in apricot for phenology and fruit quality traits [56], highlighting the complex inheritance and influence of genetic diversity.

**Table 3:** Description of each linkage group (LG) for each genitor in the F_1_ Japanese plum population ‘Red Beaut’ × ‘Santa Rosa Precoz’.

	**LG**	**SNPs**	**Total distance (cM)**	**SNP density (cM/SNP)**	**Maximum gap (cM)**
RB	1	66	89.47	1.36	16.98
	2.1	31	47.19	1.52	10.35
	2.2	7	24.54	3.51	12.95
	3	61	152.27	2.50	16.00
	4	52	116.35	2.24	10.37
	5.1	45	62.60	1.39	15.60
	5.2	12	10.08	0.84	3.01
	5.3	14	5.99	0.43	1.11
	6.1	44	64.25	1.46	20.15
	6.2	33	24.22	0.73	3.07
	7	0			
	8	63	49.29	0.78	4.05
	Total	428	646.26	1.52	
SRP	1.1	13	82.40	6.34	17.63
	1.2	17	77.11	4.54	12.03
	2.1	14	11.40	0.81	2.51
	2.2	19	25.52	1.34	7.02
	2.3	17	12.34	0.73	2.11
	3	94	158.35	1.68	12.45
	4	13	149.37	11.49	20.08
	5	53	139.37	2.63	16.01
	6	42	57.85	1.38	14.87
	7	13	105.98	8.15	13.49
	8	10	89.54	8.95	16.29
	Total	305	909.23	4.37	

Although most phenological traits showed significant differences across years, the correlation coefficients between years were consistently high. This suggests that, while environmental conditions influence phenology, as previously reported for Japanese plum [11], the strong interannual correlations point to a substantial genetic effect governing the expression of these phenological traits, despite the significant environmental influence. Salazar *et al.* [11] observed interannual correlations for bloom date in Japanese plum that were lower than those found in the present study, with Pearson correlation coefficients ranging from 0.49 to 0.59 (*P* < 0.0001). Additionally, for ripening date, the highest interannual correlation they reported ranged between 0.69 and 0.78, values that are lower than those observed in our three populations (0.959, *P*-value <0.001). This interannual variability in phenological traits is influenced by varying climatic conditions each year, particularly temperature, which affects winter chilling accumulation. The differences in chilling accumulation and its dynamic variation between years contribute to the observed phenological differences, as noted by other researchers [54, 57]. Moreover, many of the evaluated traits exhibited substantial and significant interannual correlations, further supporting the idea that genetic factors play a significant role in phenological expression, despite environmental effects.

Additionally, high correlation coefficients were observed between RD and FDP, with coefficients exceeding 0.90^***^ in the RB × BS and RB × SRP populations over the three years of evaluation. Similar correlations were reported by [14] in Japanese plum, with RD and FDP showing correlation coefficients greater than 0.75. In the RB × SRP population, flowering date (FD) was significantly correlated with RD throughout the three years. Specifically, the years 2019 and 2020 showed the highest correlations, with values of 0.44^***^ in 2019 and 0.47^***^ in 2020, suggesting that later flowering in the genotypes results in delayed ripening. This correlation has also been reported in Japanese plum by [15] in the ‘98–99’ × ‘Angeleno’ population.

To date, research on QTLs linked to phenological traits in Japanese plum species has been limited, as most studies have focused on fruit quality traits [15]. As a result, information on the genetic architecture of these phenological traits is scarce, highlighting the importance of the present study. In this study, we successfully identified a large number of QTLs associated with phenology and investigated the allelic effects of significant markers. This analysis enabled the identification of significant SNPs linked to regions associated with phenological traits, which could be used in marker-assisted selection.

The results obtained regarding the QTLs associated with flowering dates (BF, FF, and EF) across the three populations highlight the considerable complexity of this trait. In the BS × PIO population, the most statistically significant QTLs associated with BF were located in the LG3.1 of BS, as well as in LG3 and LG4 of PIO. These two candidate regions in PIO may contribute to the unique lack of correlation between BF and both FF and EF, suggesting a distinct genetic architecture underlying BF in this population. This is likely attributable to alleles inherited from PIO, which is characterized by a notably low chilling requirement—approximately 334 chill units or 22 chill portions [58]. Conversely, within the RB × BS population, the most notable QTLs for FF were identified in the LG1.3 of RB and LG6 of BS. The comparison of the QTLs identified in the four progenitors further emphasizes the diverse genetic regions involved, reinforcing the hypothesis that the expression of flowering time is influenced by multiple genes. This finding is consistent with previous studies on species such as peach, sweet cherry, and apricot [59], which also suggest that flowering time is a polygenic trait with complex genetic control. One previous study focused on identifying flowering-related QTLs in Japanese plum and successfully detected QTLs on LG1, LG6, and LG7 [15]. Furthermore, Salazar *et al.* [60] used SNP-based genome-wide association studies (GWAS) with GBS to identify markers associated with flowering date in Japanese plum. Additionally, Branchereau *et al.* [61] evaluated the traits of flowering onset, full bloom, and end of flowering in two populations of sweet cherry, detecting QTLs for these traits in nearly all linkage groups across both populations. The complex genetic control of flowering has also been clearly confirmed here, with the most significant markers located in the terminal region of LG1 and the middle region of LG7, while LG4, LG6, and LG8 also showed correlations with flowering date. Through the detection of candidate genes, this study confirms and expands previous knowledge in Japanese plum about flowering.

Regarding flowering intensity, studies identifying QTLs in *Prunus* species have been limited to date. In the present study, we have revealed the presence of QTLs associated with flowering intensity in various genomic regions, depending on the population under investigation. In the BS × PIO population, significant QTLs were identified in the PIO parent on LG4, associated with flowering onset, as well as in two segments of LG7. In the ‘RB × BS’ population, QTLs were located on LG1.1, LG2, LG4.2, LG6, and LG8.2, with the most significant QTL found on LG2 of the RB parent, overlapping with both flowering and productivity traits. The most important QTLs for BS were found on LG1.2, associated with productivity, and on LG6, linked to flowering. A previous study by Sánchez-Pérez *et al.* [54] identified a QTL for flowering intensity in almond, specifically on LG4; however, this QTL exhibited very low significance, explaining less than 12% of the phenotypic variance, and was therefore not considered a major QTL.

Concerning the identification of QTLs associated with RD and FDP, significant findings were observed across the three populations. The female parental lines—BS in BS × PIO, RB in RB × BS, and RB in RB × SRP—all exhibited highly significant QTLs for both RD and FDP on LG4. These results align with previous studies on Japanese plum [4, 15] and other *Prunus* species such as peach, apricot, and sweet cherry [59, 62], where LG4 has consistently been identified as a crucial genomic region regulating these traits. In contrast, the male parental lines displayed a greater diversity of linkage groups with significant QTLs, with the most notable being two segments of LG1 (LG1.1 and LG1.2) and LG2 in PIO, LG3.3 and LG5 in BS, and LG1.1 in SRP. Furthermore, it is noteworthy that, across most linkage groups, the QTLs associated with RD and FDP often exhibit spatial overlap, as indicated by their shared confidence intervals.

Regarding productivity, QTLs were identified in different genomic regions, depending on the specific population. In the BS × PIO population, the most significant QTL was located on LG1, while in the PIO parent, QTLs associated with productivity were found on LG5. In the RB × BS population, the most significant QTLs were located on LG2 and LG4.1 in RB, while in BS, the second segment of LG1 showed the most notable QTLs, similar to the findings in the first population. The RB × SRP population was the only one where no consistent QTLs were detected across the three years, perhaps due to some limitation in the genetic architecture caused by the genetic origin of both parents. Variability in the localization of QTLs across linkage groups has also been reported in other studies. Sánchez-Pérez *et al.* [54] identified a QTL for productivity on LG4 in almond, though its significance was highly irregular, explaining a very low percentage of the phenotypic variance. In peach, QTLs related to productivity have been identified in various linkage groups, such as LG8 [63] and LG6 [64]. These findings, therefore, underscore the complexity and variability of the genetic factors influencing productivity in *Prunus* species.

The prediction of the effects of all significant SNPs associated with the most conserved QTLs (present in at least two years) was performed using SnpEff [65]. The 95 unique SNPs produced a total of 236 predicted effects. Of these, 212 (89.83%) had modifier impact, 11 (4.66%) had moderate impact, and 13 (5.51%) had low impact (Table S21). The largest number of effects was detected for full flowering (FF) time, with 62 effects. A list of candidate genes associated with our target traits was identified. Some can be considered priority candidates for marker development, such as ethylene receptors, heat shock transcription factors, and shikimate hydroxycinnamoyl transferase (HCT). The enzyme shikimate O-hydroxycinnamoyl transferase [EC:2.3.1.133] was affected by an SNP located in a stable QTL for BF and plays a critical role in the phenylpropanoid pathway during seed-plant development [66]. In transgenic alfalfa lines in which HCT levels were severely down-regulated, significant stunting, reduced biomass, and delayed flowering were observed [67].

For FF, significant SNPs affected several ethylene receptors, for example ethylene receptor [EC:2.7.13.-], and EREBP-like factors. EREBPs, together with AP2 (APETALA2), compose the AP2/EREBP superfamily, one of the largest groups of plant-specific transcription factors. They play vital roles in plant growth and development and in responses to diverse stresses including extreme temperatures, drought, high salinity, and pathogen infection [68]. Other enzymes affected by significant SNPs were methionine-cycle or MTA (Yang cycle) enzymes, specifically 5-methylthioribose kinase and methionine synthase, which supply ethylene precursors [69, 70]. In addition, Rab11A-dependent tip-focused trafficking and acyl-CoA synthetase functions provision sporopollenin and lipid-derived aroma and support pollination and volatile emission [71, 72].

For EF, the same significant SNP S8_27668999 detected for FF affected the ethylene receptor or EREBP-like factor. In addition, another significant SNP affected the regulator of nonsense transcripts 3 (UPF3). In *Arabidopsis*, loss of the core NMD proteins UPF1 and UPF3 leads to late flowering, which suggests that the nonsense-mediated mRNA decay surveillance system can epigenetically modulate flowering time [73].

A common significant SNP across all flowering dates, S4_28542930, located in a stable QTL on chromosome 4, had a modifier effect on carboxy-terminal domain (CTD) RNA polymerase II polypeptide A small phosphatase [EC:3.1.3.16]. This CTD small phosphatase dephosphorylates the RNA polymerase II C-terminal domain. CTD small phosphatases, such as CPL1 and CPL3, act as regulatory switches in flowering control by modulating CTD phosphorylation and by interacting with key flowering repressors such as FLOWERING LOCUS C (FLC) [74–76]. Through these actions, CTD small phosphatases integrate signals that finely regulate flowering time and ensure flowering at appropriate developmental stages and under suitable environmental conditions [74–76]. In *Prunus* species, especially Japanese plum (*P. salicina*), there is no previous evidence that specifically links CTD phosphatases to flowering regulation as described in *Arabidopsis*.

For Productivity, the significant SNP S1_27714189 showed an effect on urease [EC:3.5.1.5], a nickel enzyme that hydrolyzes urea into ammonia and carbamate, which then forms carbonic acid and ammonia. In perennial fruit trees such as apple and peach, urease activity hydrolyzes foliar-applied urea into usable nitrogen. This enhances nitrogen remobilization, vegetative growth, and ultimately fruit yield efficiency [77, 78].

For ripening date, the significant SNP S1_39702792 affected a heat shock transcription factor. Heat shock transcription factors regulate the expression of heat shock proteins in response to diverse stimuli [79], including cold, drought, and salinity, and during developmental processes such as embryogenesis, germination, and fruit development [80–82]. Same significant SNP affected an carlactone C-19 oxidase [EC:1.14.-.-] enzyme. This enzyme belongs to the cytochrome P450 family, specifically a MAX1 homolog, that oxidizes carlactone (CL) to carlactonoic acid, a key step in strigolactone biosynthesis. In woodland strawberry (a non-climacteric fruit), strigolactone biosynthetic and signaling genes—including MAX1 homologs—are highly expressed during early fruit development but decline sharply at ripening onset, and low or no expression was detected in ripening fruits [83].

Another significant SNP for RD, S1_53963445, affected chloride channel 7 (CLCN7). The CLC family is expressed on internal membranes of plant cells and functions as essential chloride exchangers or channels. Although no studies in Japanese plum or other fruit species currently implicate CLCN7 directly in ripening, CLCs play key roles in ion homeostasis, osmotic regulation, and vacuolar storage. Moreover, chloride can enhance sugar metabolism and fruit sweetness in tomato by stimulating specific metabolic enzymes during fruit development and ripening [84].

Finally, although additional studies are required to rigorously demonstrate SNP–trait associations in Japanese plum, the candidate-gene information reported here provides a useful resource for the *P. salicina* research community and may guide future research in this species. In the case of flowering intensity or fruit development period (FDP) the candidates seems of lower-priority and additional studies must be completed to better understand the genetic complexity of these traits.

## Materials and methods

### Plant material

The plant material assayed included three F_1_ progenies from intraspecific crosses of four Japanese plum cultivars: ‘Black Splendor’ × ‘Pioneer’ (BS × PIO); ‘Red Beaut’ × ‘Black Splendor’ (RB × BS) and ‘Red Beaut’ × ‘Santa Rosa Precoz’ (RB × SRP) with 121, 103, and 103 seedlings, respectively (Fig. 1). All parental trees were grafted onto the commercial rootstock Mariana 2624, whereas the progenies were grown on their own root. Pedigrees of the parental genotypes are shown in Fig. 2, and their main agronomic traits are summarized in Table S1. The crosses followed the standard methodology of a classical breeding program, including controlled pollination under mesh in the field, embryo rescue, greenhouse acclimation, and subsequent field plantation. Crosses were performed as part of the Japanese plum breeding program developed jointly by CEBAS-CSIC and IMIDA of Murcia (Spain). The RB × BS and RB × SRP seedlings were planted on field conditions in 2013, while BS × PIO progeny was planted two years later. All seedlings were cultivated in the same experimental orchard located in Calasparra (Murcia, southeastern Spain; lat. 38°16′N, long. 1°35′W; 350 m altitude) according to standard Japanese plum orchard management.

### DNA extraction

Young leaves were sampled during the spring season and preserved at −80°C until DNA extraction. The DNA isolation process was conducted following a modified version of the CTAB method initially outlined by [26]. The DNA concentration was precisely quantified by Qubit 2.0.

#### Whole genome sequencing and assembly of parental genomes

Parental genomes were sequenced at ~80× coverage by Novogene using Illumina paired-end technology. Libraries were prepared via fragmentation, adapter ligation, PCR amplification, and quality-checked using a Bioanalyzer and qPCR. *De novo* assembly was performed with MaSuRCA [27], and assembly quality was evaluated with QUAST [28]. Read alignment back to the assemblies was done using Bowtie2 [29], and genome completeness was assessed using BUSCO v5 with the embryophyta_odb10 dataset [30].

Variant calling was performed by aligning trimmed reads to the *P. salicina* cv. Sanyueli v2.0. genome using BWA-MEM [31]. SAM files were converted to BAM, sorted, and indexed with SAMtools [32] and Picard tools [33]. Variants were called using BCFtools v1.1356 [32] and filtered with the following thresholds: QUAL > 19 && DP > 2$ && (AC/AN) > 0.05 && MQ > 20.

### Calling SNPs on progeny from reduced-representation Illumina data

Genotyping-by-sequencing (GBS) libraries were prepared in 96-well plates using simultaneous restriction-ligation with either HindIII-HF or PstI-HF in combination with MseI and T4 DNA Ligase (New England Biolabs, Frankfurt) [34]. Individual barcoded libraries were pooled by 96-well plate, cleaned using Ampure XP beads, PCR amplified, and then cleaned again with Ampure XP beads before loading onto an Agilent Bioanalyzer 2100 in a DNA7500 chip. Libraries were adjusted to 10 nmol before submission to the UC Davis Genome Center for SR100 sequencing on a HiSeq4000 instrument. All the data were in a single Illumina HiSeq lane. The TASSEL GBS pipeline [35] and BWA [31] were used to align 64 bp tags to the *P. salicina* cv. ‘Sanyueli’ assembly [20], retaining only tags with a BWA MAPQ score of at least 20. Subsequently, SNPs were filtered separately for each parent in each population using the corresponding genome assembly. Only assembly SNPs supported with a depth of at least 8X in both parents were considered, and only SNPs heterozygous in the focal parent and homozygous in the other parent were included in the filtered SNP list for each parent of each population. The filtered SNP lists (eight in total) were used as input for FSFHap (Full-Sib Family Haplotype Imputation) [36] to correct for under-calling of heterozygous genotypes, and FSFHap output was used for linkage map construction.

### Evaluation of phenological traits and data analysis

The phenological traits of interest, including the beginning of flowering (BF), full flowering (FF), end of flowering (EF), flowering intensity (FI), ripening date (RD), fruit development period (FDP) and productivity (P), were evaluated for three consecutive years (2019, 2020, and 2021) in the offspring and their respective parents. The assessment of flowering time was conducted periodically in the orchard at 3–4 day intervals, with results expressed in Julian days. BF was determined when 5% of the flowers had opened, FF was recorded at stage 65 according to the BBCH scale [37] when 50% of the flowers had opened, and EF was recorded when 90% of the flowers had opened. FI was quantified on a scale from 0 to 3. Additionally, RD was determined at physiological maturity, characterized by appropriate fruit firmness and color, while P was visually scored on a scale from 0 (null) to 5 (maximum). Finally, FDP was calculated as the number of days between FF and RD.

Statistical analyses were performed using IBM SPSS (v27) [38]. Frequency histograms were generated to show the distribution of seedlings for each trait, year, and population. The normality of all phenotypic datasets was tested using the Kolmogorov–Smirnov test with Lilliefors correction, considering genotype and year as independent factors. Traits that did not meet the normality criteria were analyzed using the non-parametric Kruskal–Wallis test. Additionally, Spearman correlation coefficients were calculated to assess the relationships between traits within the same year and the interannual correlations for each trait, using raw data and a significance level of *P* ≤ 0.05 for the three populations. Principal components analysis (PCA) was performed on the phenological data to facilitate visualization of the complete dataset in a reduced-dimensional plot, thereby identifying related groups, trends, or outliers. Spearman correlations, PCA calculations, and plotting were carried out using the R packages ‘corrplot’ [39], ‘factoextra’ [40], and ‘FactoMineR’ [41].

### Linkage map construction and quantitative trait loci analysis

Genetic linkage maps were constructed for each parent in the BS × PIO, RB × BS, and RB × SRP populations using JoinMap® 4.1 [42]. SNP markers heterozygous in one parent and homozygous in the other (<lmxll> or < nnxnp>) were used. Markers with >40% missing data, significant segregation distortion (χ^2^, *P* < 0.001), or complete redundancy (Similarity of Loci = 1) were excluded. Mapping employed the Maximum Likelihood algorithm with Haldane’s function, a recombination frequency threshold of 0.4, and LOD scores between 6 and 10. SNP positions were based on the *P. salicina* ‘Sanyueli’ v2.0 genome [16]. Six parental maps were generated independently, with no consensus map constructed. QTL analysis was performed in MapQTL® 6 [43] using the Kruskal–Wallis non-parametric test and 1000 permutations to define trait-specific significance thresholds (α < 0.05, 0.01, 0.001). Stable QTLs were defined as those consistently detected with *P* < 0.05 across all three years. The most significant markers within these QTLs were further analyzed for genotype–phenotype associations, assessed by Kruskal–Wallis tests and visualized with the R packages ‘LinkageMapView’ [44] and ‘ggplot2’ [45].

## Conclusions

These findings provide valuable genetic and genomic resources for Japanese plum and identify strong candidate markers for the future application of marker-assisted selection in breeding programs. This study presents an innovative and cost-effective strategy for QTL identification related to phenological traits in *Prunus*, based on the integration of high-coverage and low-coverage whole-genome sequencing (lcWGS) in both parental and progeny populations. Trait correlations, together with principal component analysis, offered meaningful insights into the relationships among phenological traits and the genetic mechanisms underlying their expression. Genetic linkage maps were successfully developed for all parental lines across three populations, enabling efficient QTL detection at a significantly lower cost than traditional approaches such as AFLP, GBS, or SLAF-seq. Notably, the most significant SNPs showed consistent and stable allelic effects across three years, reinforcing their potential as molecular markers to support future breeding in Japanese plum. In addition, we nominate a biologically plausible set of candidate genes within stable QTLs as testable hypotheses. These candidates require genetic and functional validation in *P. salicina*, but they provide immediate targets for functional studies and future marker development for Japanese plum breeding.

## Supplementary Material

Web_Material_uhaf271
