# Supplementary figures and images for: Identification of phenological QTLs using a combination of high- and low-coverage whole genome sequencing in Japanese plum (Prunus salicina Lindl.)
Source: Hortic Res. 2025 Nov 4;13(1):uhaf271. doi: 10.1093/hr/uhaf271 (PMC12863216; doi:10.1093/hr/uhaf271)

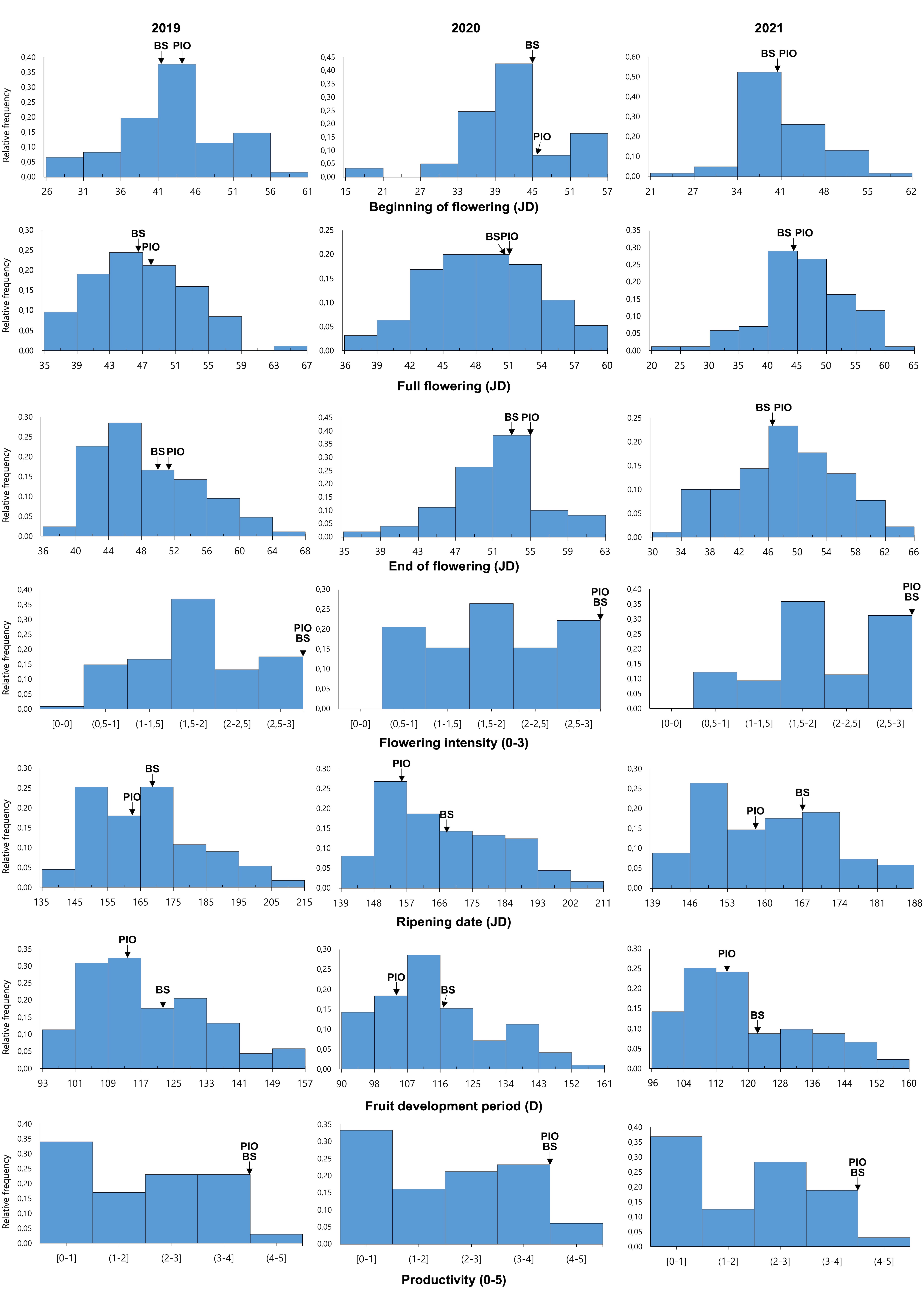

Supplement: Web_Material_uhaf271 [file web_material_uhaf271.zip › Figure S1. Histograms BSxPIO-modificada.jpg]

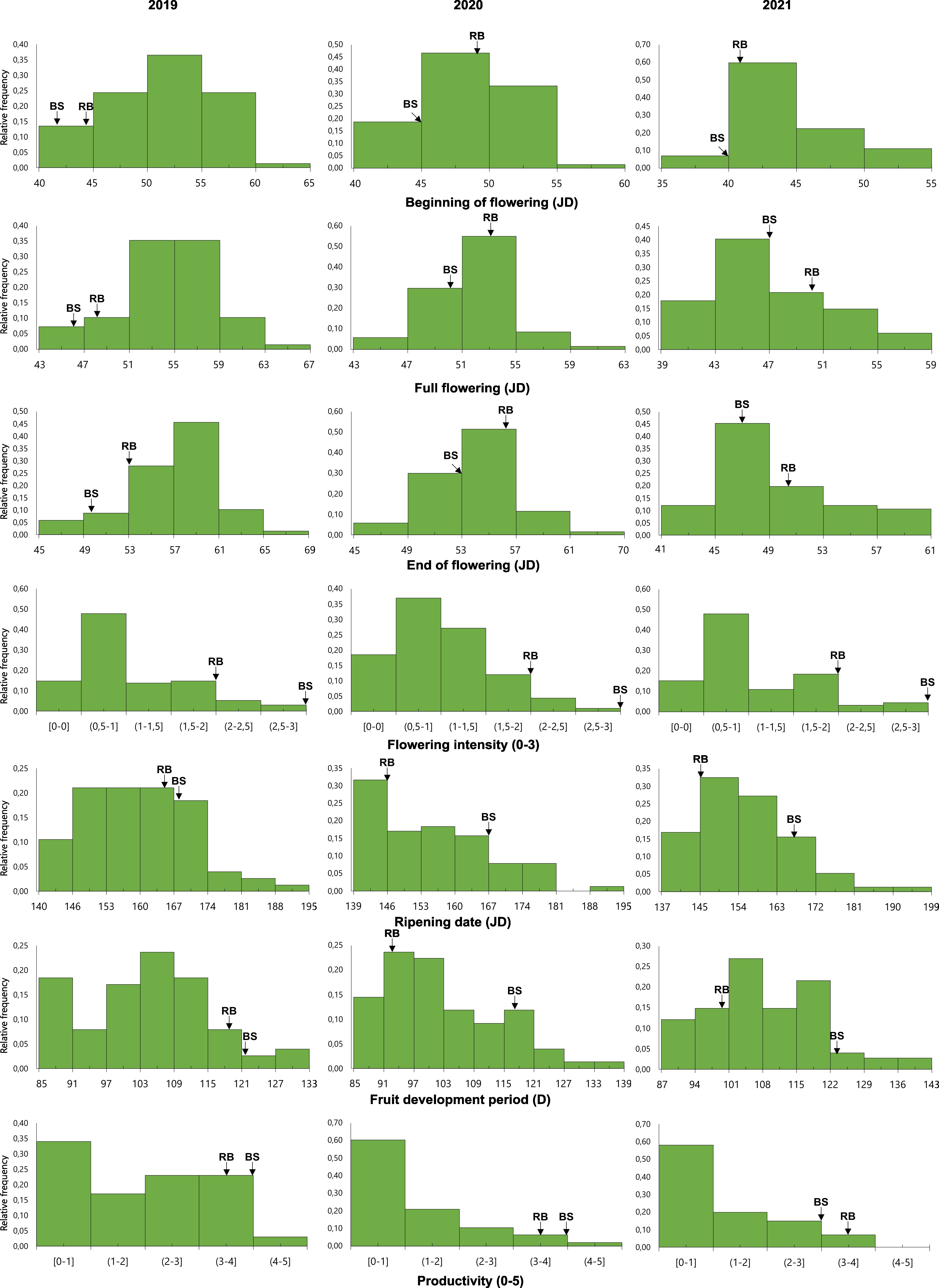

Supplement: Web_Material_uhaf271 [file web_material_uhaf271.zip › Figure S2. Histograms RBxBS.jpg]

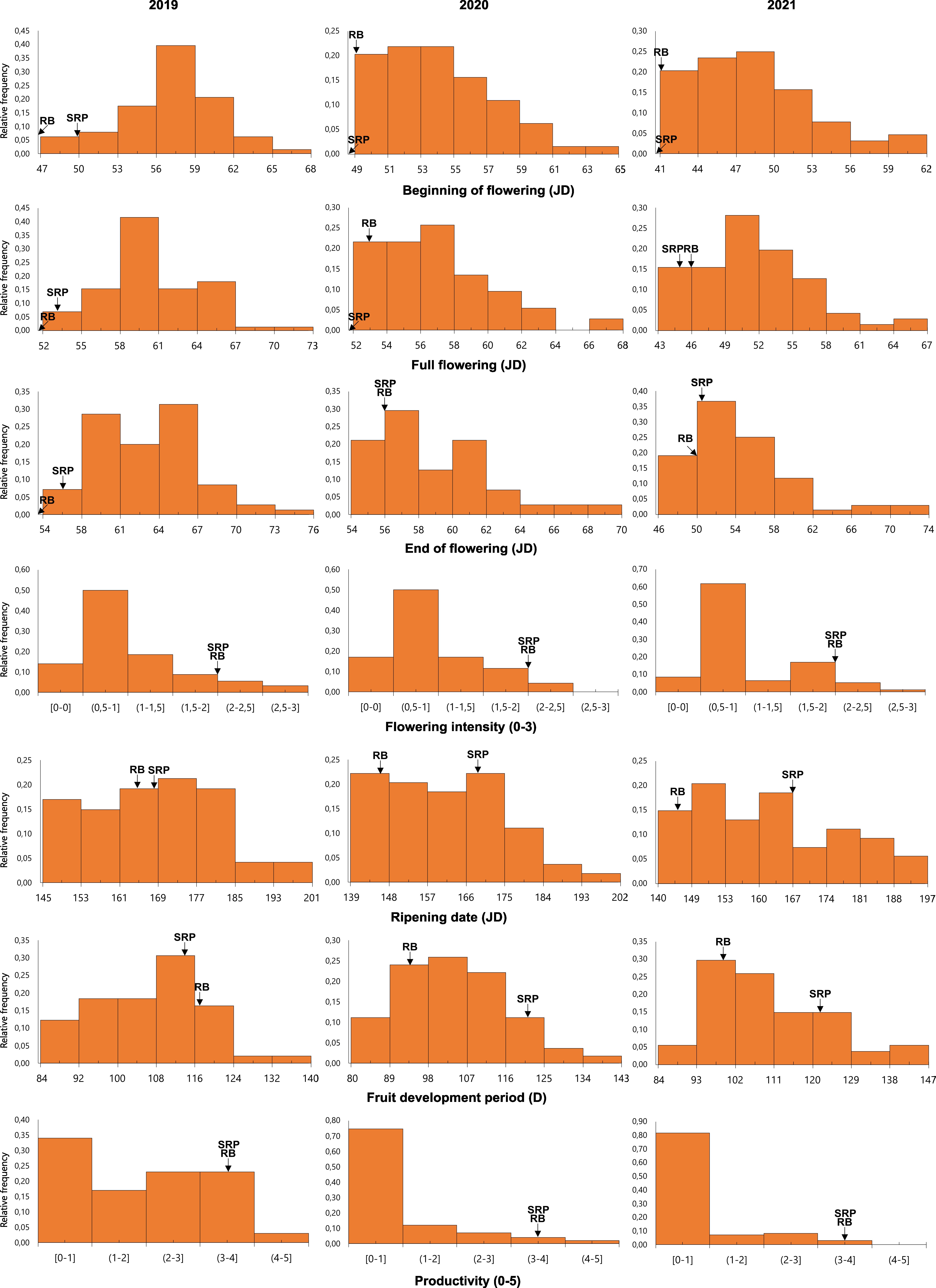

Supplement: Web_Material_uhaf271 [file web_material_uhaf271.zip › Figure S3. Histograms RBxSRP.jpg]

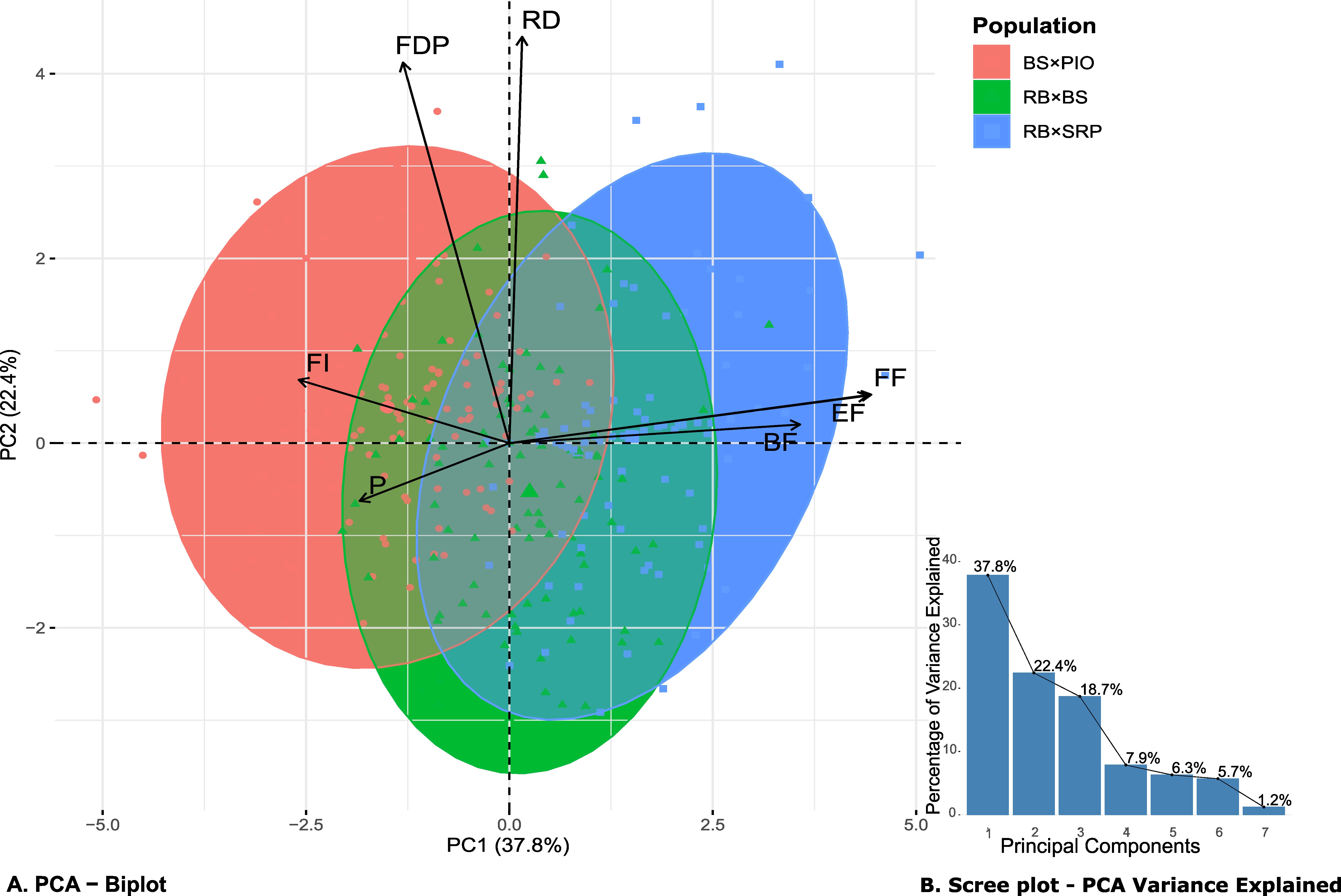

Supplement: Web_Material_uhaf271 [file web_material_uhaf271.zip › Figure S4. PCA, Screeplot.jpg]

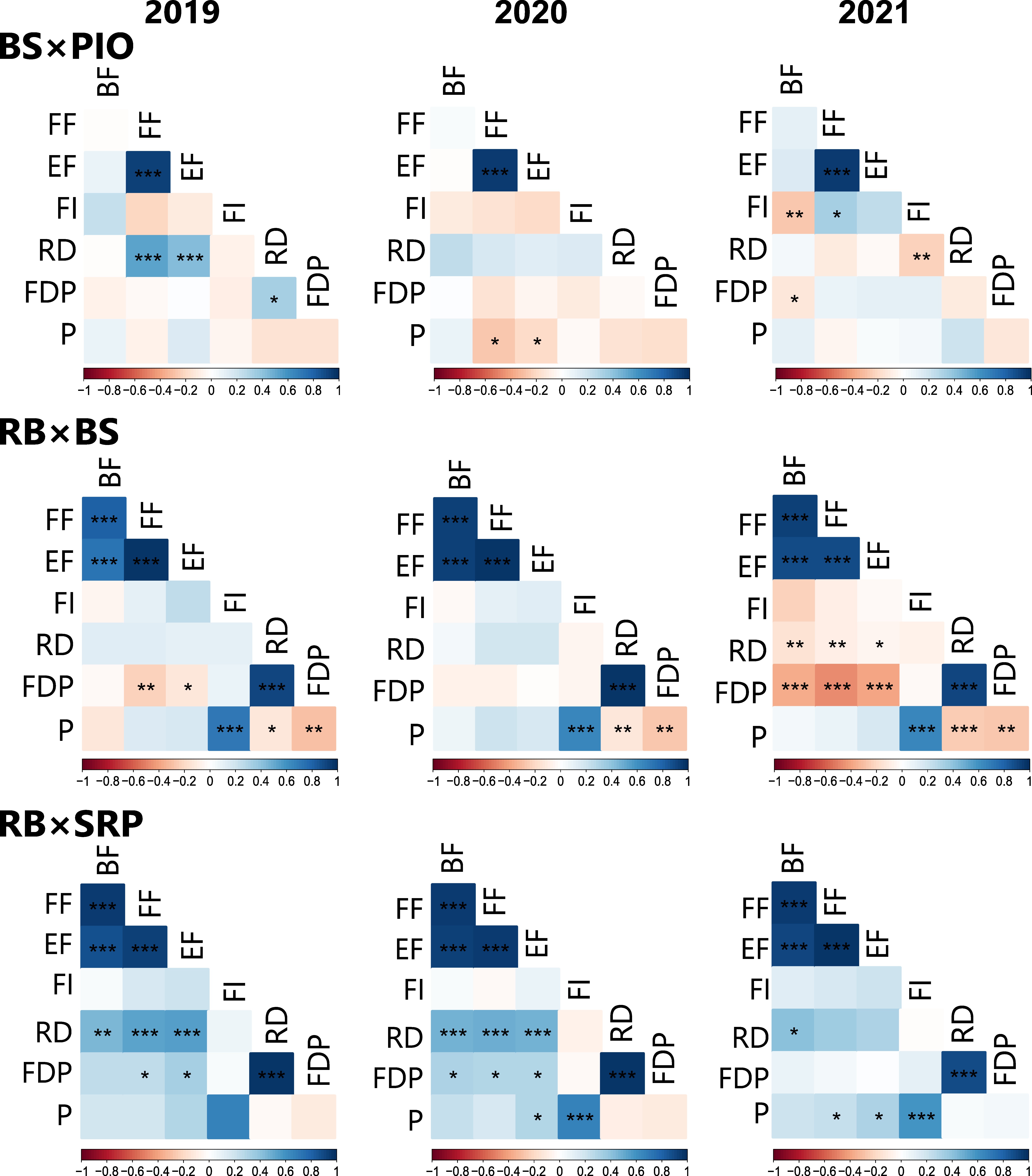

Supplement: Web_Material_uhaf271 [file web_material_uhaf271.zip › Figure S5. Spearman correlations-modificada.jpg]

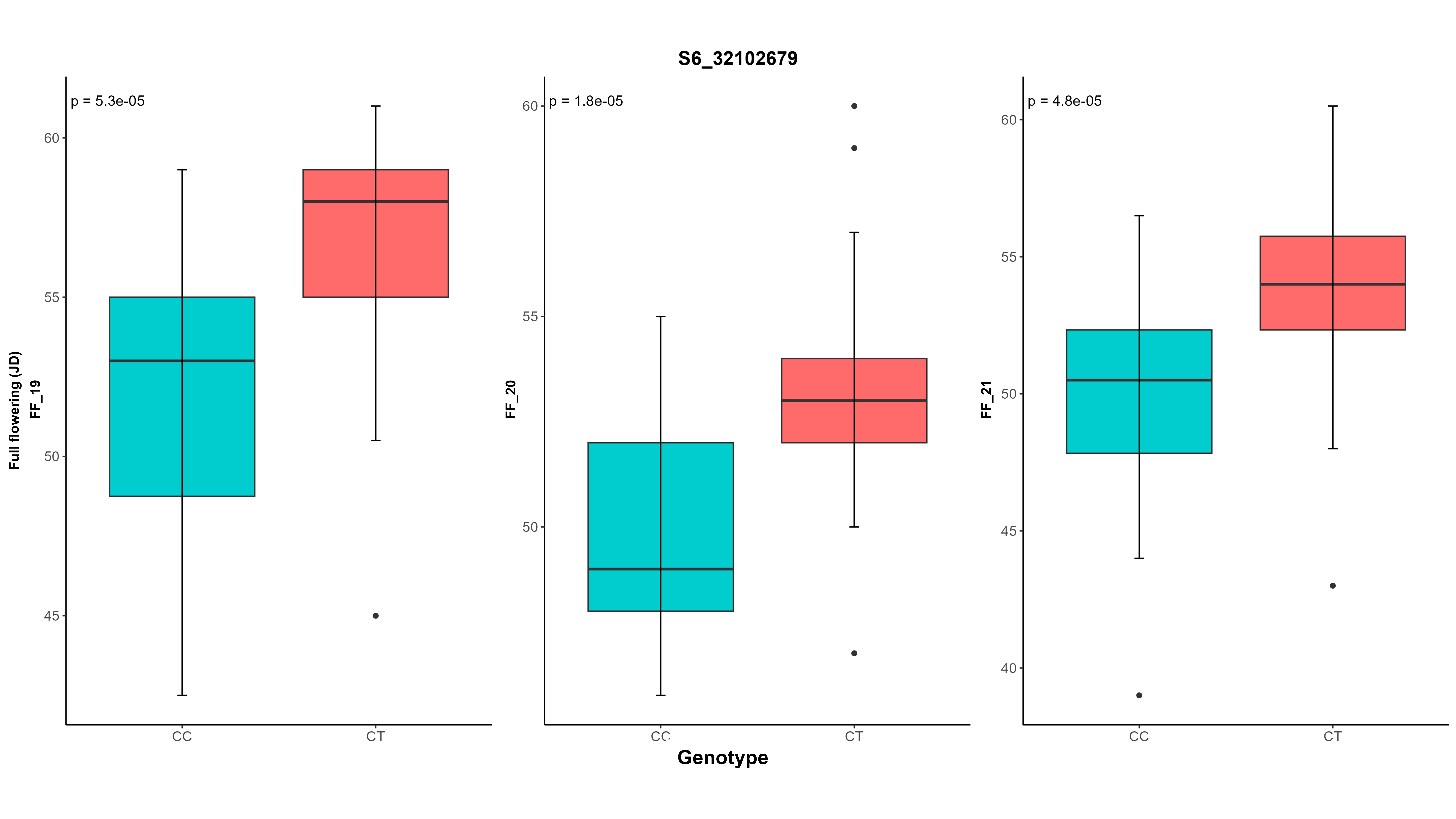

Supplement: Web_Material_uhaf271 [file web_material_uhaf271.zip › Figure S6.jpg]

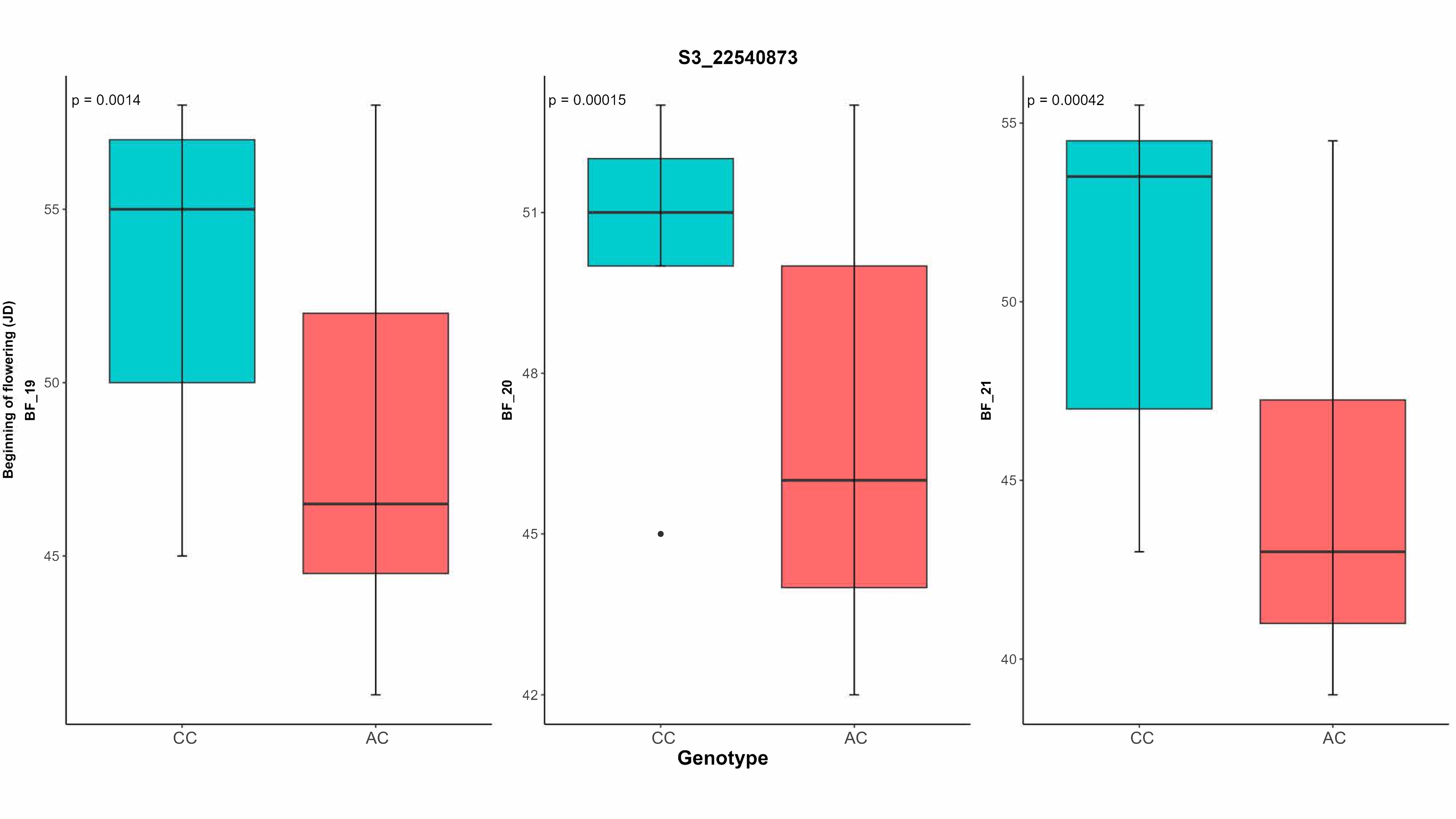

Supplement: Web_Material_uhaf271 [file web_material_uhaf271.zip › Figure S7-new.jpg]

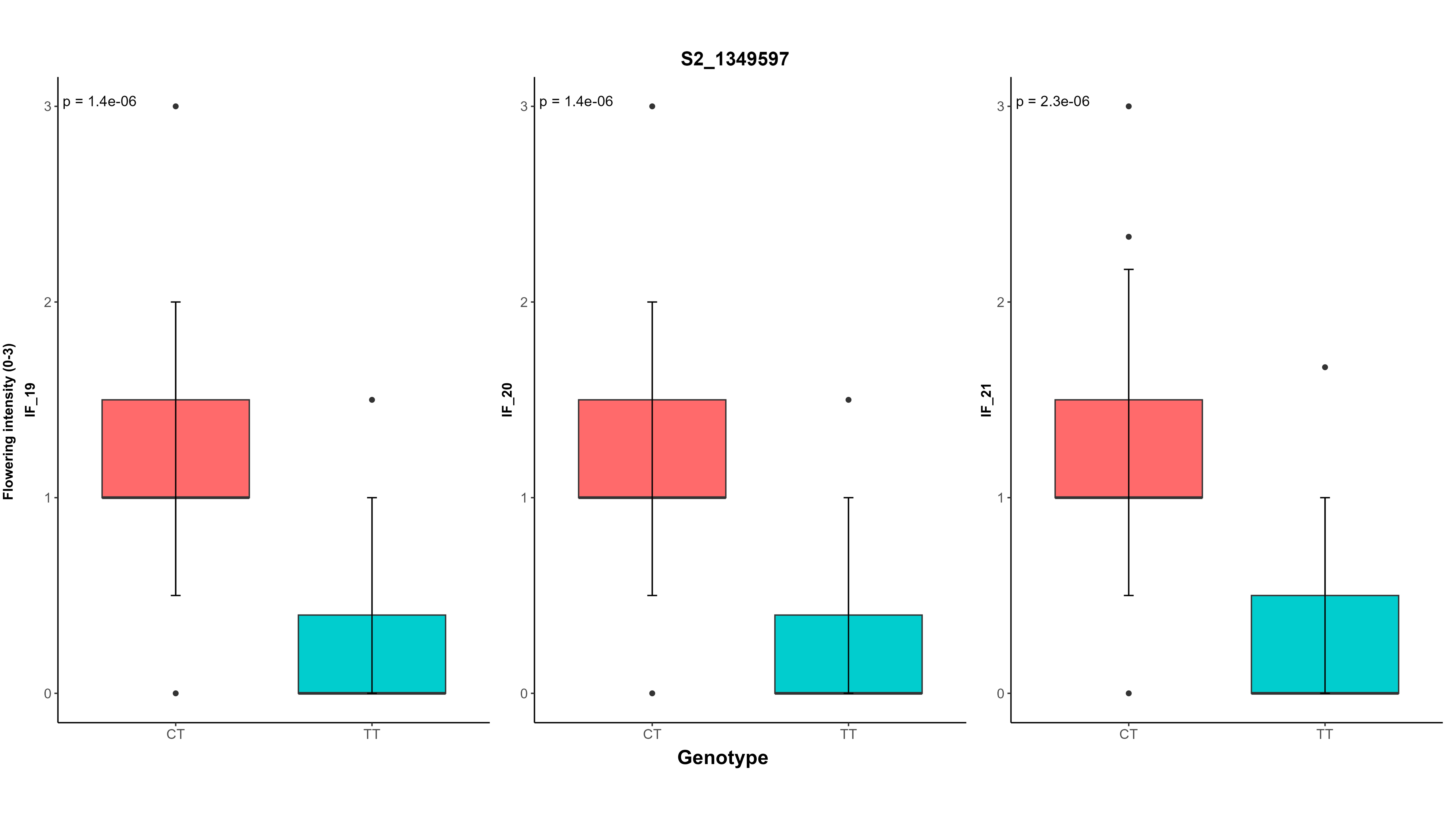

Supplement: Web_Material_uhaf271 [file web_material_uhaf271.zip › Figure S8.jpg]

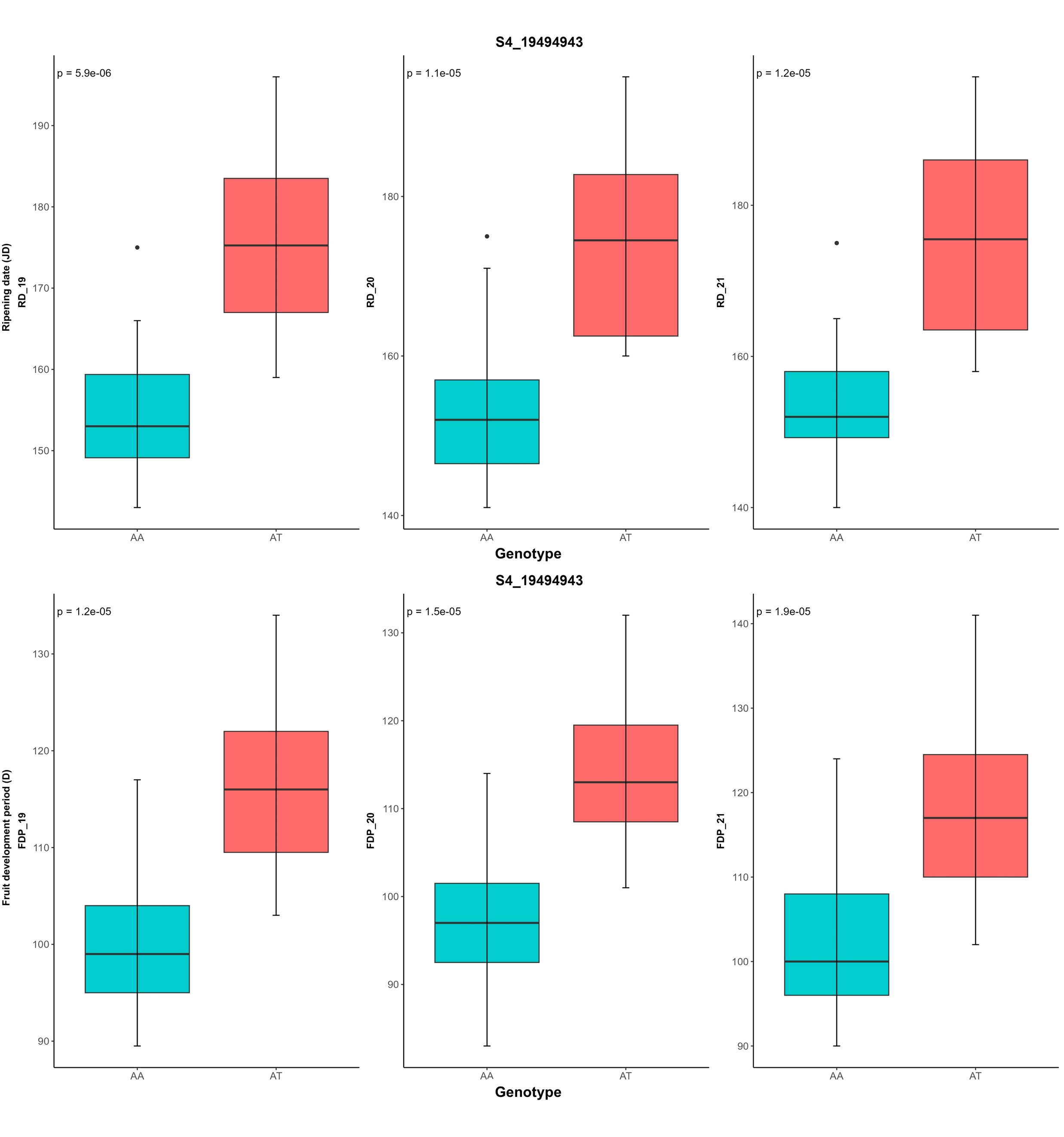

Supplement: Web_Material_uhaf271 [file web_material_uhaf271.zip › Figure S9.jpg]
